# Examining the Research Criteria for Traumatic Encephalopathy Syndrome in Middle-Aged Men From the General Population Who Played Contact Sports in High School

**DOI:** 10.3389/fneur.2021.632618

**Published:** 2021-04-14

**Authors:** Grant L. Iverson, Zachary C. Merz, Douglas P. Terry

**Affiliations:** ^1^Department of Physical Medicine and Rehabilitation, Spaulding Rehabilitation Hospital, Charlestown, MA, United States; ^2^Department of Physical Medicine and Rehabilitation, Harvard Medical School, Boston, MA, United States; ^3^Spaulding Research Institute, Charlestown, MA, United States; ^4^Sports Concussion Program, MassGeneral Hospital for Children, Boston, MA, United States; ^5^Home Base, A Red Sox Foundation and Massachusetts General Hospital Program, Charlestown, MA, United States; ^6^LeBauer Department of Neurology, Moses H. Cone Memorial Hospital, Greensboro, NC, United States

**Keywords:** assessment, head injury, traumatic brain injury, chronic pain, depression, concussion

## Abstract

**Objective:** There are no validated or agreed upon diagnostic clinical criteria for chronic traumatic encephalopathy or traumatic encephalopathy syndrome. This study examines the leading research criteria for traumatic encephalopathy syndrome (TES) in middle-aged men in the general population.

**Method:** Participants were 409 men between the ages of 35 and 55 recruited through an online crowdsourcing platform. Participants provided demographic information, medication history, concussion history, contact sport history, current medication use, and current symptoms. Research criteria for TES were applied to the sample.

**Results:** Over half of the total sample met TES symptom criteria (56.2%), without applying the neurotrauma exposure criteria. Those with 4+ prior concussions had higher rates of meeting TES criteria compared to those with 0–3 prior concussions, but the results were not statistically significant (69.8 vs. 54.6%; χ^2^ = 3.58, *p* = 0.06). Exposure to contact sports was not related to higher rates of TES (ps ≥ 0.55). In a binary logistic regression predicting the presence of mild or greater TES, significant predictors were sleep difficulties [Odds ratio (OR) = 6.68], chronic pain (OR = 3.29), and age (OR = 1.04). Neurotrauma exposure was not a significant predictor (*p* = 0.66). When analyzing those with no prior concussions or contact sport histories (*n* = 126), 45.2% met symptom criteria for mild or greater TES; chronic pain and sleep difficulties were associated with a higher prevalence of meeting criteria for TES in this subgroup (ps < 0.001).

**Conclusions:** Men who participated in contact sports in high school or college were not more likely to meet criteria for TES than men who participated in non-contact sports or no sports. In a multivariable model, sleep problems and chronic pain were predictive of meeting the symptom criteria for TES, but the repetitive neurotrauma exposure criterion was not a significant predictor of meeting the TES symptom criteria.

## Introduction

In the twentieth century, chronic traumatic encephalopathy (CTE) was conceptualized as a neurological syndrome associated with cumulative brain damage from a long career in boxing (1, 2). Throughout the century, it was also referred to as dementia pugilistica (3–7). It was typically described as Parkinsonian-like in nature, with dysarthric speech, gait and coordination difficulties, and cognitive deficits (1, 2, 5). Some authors conceptualized it as a progressive dementia and others noted that it could have a static or progressive course (1, 2, 4, 7–13). The authors of case studies and case series in the twentieth century also described what appeared to be neuropsychiatric changes in personality or behavior, such as aggression and volatility (11, 14–18), euphoria (9, 16), child-like behavior (17), or fatuous cheerfulness (16).

Over the past 100 years, there have been no validated or agreed upon diagnostic clinical criteria for CTE, sometimes referred to as traumatic encephalopathy syndrome (TES). Several sets of criteria were proposed between 2013 and 2016 (12, 20–22). A team of investigators from Boston University proposed criteria to be used for identifying TES in living research participants (19), and these research criteria are reprinted in Table 1. These research criteria are being used in studies included in a $15.8M multicenter grant entitled “Diagnostics, Imaging, And Genetics Network for the Objective Study and Evaluation of Chronic Traumatic Encephalopathy” (DIAGNOSE CTE; NIH/NINDS Grant No. U01NS093334). Moreover, in April of 2019, this research team hosted the First NIH Consensus Workshop to Define the Diagnostic Criteria for Traumatic Encephalopathy Syndrome (TES), and the work to establish consensus criteria has been ongoing.

**Table 1:** Research criteria for Traumatic Encephalopathy Syndrome (core and supportive features) (19).

**Core Clinical Features**
“At least one of the core clinical features must be present: 1. *Cognitive*. Difficulties in cognition: (a) as reported by self or informant, by history of treatment, or by clinician's report of decline, and (b) substantiated by impairment on standardized mental status or neuropsychological tests of episodic memory, executive function, and/or attention, as defined by scores at a level of at least 1.5 standard deviations below appropriate norms. 2. *Behavioral*. Being described as emotionally explosive (for example, having a “short fuse” or being “out of control”), physically violent, and/or verbally violent, as reported by self or informant, by history of treatment, or by clinician's report. A formal diagnosis of intermittent explosive disorder would meet this criterion but is not necessary. 3. *Mood*. Feeling overly sad, depressed, and/or hopeless, as reported by self or informant, by history of treatment, or by clinician's report. A formal diagnosis of major depressive disorder or persistent depressive disorder would meet this criterion but is not necessary.” [(19), p. 10]
**Supportive Features**
“A minimum of two of the supportive features must be present for a diagnosis of TES: 1 *Impulsivity*. Impaired impulse control, as demonstrated by new behaviors such as excessive gambling, increased or unusual sexual activity, substance abuse, or excessive shopping or unusual purchases, or similar activities. 2* Anxiety*. History of anxious mood, agitation, excessive fears, or obsessive or compulsive behavior (or both), as reported by self or informant, history of treatment, or clinician's report. A formal diagnosis of anxiety disorder would meet this criterion but is not necessary. 3 *Apathy*. Loss of interest in usual activities, loss of motivation and emotions, and/or reduction of voluntary, goal-directed behaviors as reported by self or informant, history of treatment, or clinician's report. 4 *Paranoia*. Delusional beliefs of suspicion, persecution, and/or unwanted jealousy. 5 *Suicidality*. History of suicidal thoughts or attempts, as reported by self or informant, history of treatment, or clinician's report. 6 *Headache*. Significant and chronic headache with a least one episode per month for a minimum of 6 months. 7 *Motor signs*. Dysarthria, dysgraphia, bradykinesia, tremor, rigidity, gait disturbance, falls, and/or other features of parkinsonism. 8 *Documented decline*. Progressive decline in function and/or a progression in symptoms and/or signs … for a minimum of 1 year. 9 *Delayed onset*. Delayed onset of clinical features after significant head impact exposure, usually at least 2 years and in many cases several years after the period of maximal exposure. It should be noted, however, that individual cases may begin to develop the clinical features of TES during their period of head impact exposure (for example, while still actively involved in a collision sport), especially older individuals or those who have been engaged in the high-exposure sport for many years.” [(19), p. 10–11].

*Reprinted from Montenigro et al. (19)*.

These research diagnostic criteria departed substantially from how CTE and TES were conceptualized in the twentieth century in that they were broadened to include a wide range of mental health problems. In fact, mental health problems alone, such as depression, anxiety, and suicidality, were now considered sufficient symptoms for diagnosing TES (assuming the neurotrauma criteria were met). According to the research diagnostic criteria, depression is considered to be a core subtype of TES. A recent study examined the prevalence of several features of the research criteria for TES in a sample of 101 men from the US general population who were diagnosed with major depressive disorder in the past month (23). Approximately half of the sample (52.5%) met a conservative classification of TES using the symptom criteria for the mood subtype, and 8 out of 10 (83.2%) met liberal symptom criteria for this TES subtype. A second study examined the symptom criteria for the “behavioral” subtype of TES and applied those criteria to a sample of 206 men from the US general population who were diagnosed with intermittent explosive disorder in the past year, finding that 1 in 4 of these men (27.3%) met symptom criteria for the behavioral subtype of TES, and 2 out of 3 (65.0%) met liberal symptom criteria for the behavioral subtype of TES (24). Those two studies illustrated that the symptoms of TES are common in men from the general population who experience clinically significant depression or anger control problems, but they were seriously limited by the fact that they relied on a large, pre-existing epidemiological database that did not include several features of the proposed TES diagnostic criteria (e.g., cognitive impairment or neurological problems), nor did it include information relating to sport participation or neurotrauma history.

The authors of another study retrospectively reviewed the charts of veterans with a history of traumatic brain injury (TBI) who presented to a neurobehavioral clinic with cognitive complaints (21). Using the symptoms patients reported during their visits, the authors determined the prevalence of possible CTE/TES based on four different sets of proposed clinical diagnostic criteria from different research groups. Approximately 25–30% of the sample met the Montenigro et al. TES criteria (19), and 79% of the TBI-exposed sample met at least one of the four proposed sets of TES criteria. The authors reported that “these criteria mostly favor sensitivity over specificity, and can therefore result in false positives when used in a clinical setting” [(21), p. 437].

The present study examines the research criteria for TES in middle-aged men in the United States general population. This is the first study, to our knowledge, designed to sample both the proposed symptoms of TES as well as participants' prior neurotrauma histories based on their participation in contact and collision sports and their concussion history. We hypothesized that a sizable proportion of the sample would meet the proposed criteria for TES, including those who denied a history of prior concussions and a history of participation in contact sports.

## Materials and Methods

### Participants and Procedures

Participants were men between the ages 35 and 55 who are current U.S. residents. They were recruited using the online crowdsourcing platform Amazon Mechanical Turn (mTurk), which has previously been used in a variety of psychological studies (25). This platform has become increasingly common over the past 10 years and has made collecting data from large and diverse samples more accessible (26). MTurk workers are thought to be more diverse than samples commonly used in survey research studies [e.g., community samples, undergraduates; (25)]. We set a recruitment goal of 400 participants.

Institutional Review Board approval was obtained from the university prior to data collection. Participants read the study objectives and rationale and gave informed consent to participate by voluntarily completing the survey on the mTurk platform. Participants were able to withdraw from this study at any time. The survey took ~15 min to complete. Financial compensation of $5.00 was provided to survey completers. Several validity items were embedded into the survey to capture random responding. Only surveys that completed these validity items in an accurate manner were retained for data analyses.

### Survey

Participants completed a series of questions that captured demographic variables, self-reported medication history, and current medication use. Specifically, they were asked about their participation in various non-contact, contact, and collision sports. For each sport, participants were asked to provide an estimation (in years) for the following competition level categories: (1) before high school, (2) during high school, (3) during college, (4) recreationally, (5), semi-professionally, and (6) professionally. Participants also reported the number of concussions they experienced over their lifetime based on the following definition that was published in the survey: “*We define a concussion as a blow to the head or whiplash that caused any one or more of the following: (1) witnessed loss of consciousness (being “knocked out” and someone seeing it), (2) loss of memory for events immediately before and/or after the injury, or (3) feeling dazed and confused for at least 30 seconds.”* For those participants who reported a concussion, they were asked to estimate how much time had elapsed since their most recent concussion. Finally, participants were asked to rate a series of symptoms that were generated based on the TES research criteria published by Montenigro et al. (19) (see Table 1). Each symptom was rated over the past year because the research criteria indicate that the clinical features must be present for at least 12 months. Each symptom was rated on a 5-point scale (1 = Never, 2 = Rarely, 3 = Sometimes, 4 = Often, 5 = Always). Other symptoms were also assessed in this manner that are not a part of the TES research criteria [e.g., I have had pain in one or more parts of my body, I have had back or neck pain, I have had trouble falling or staying asleep, I have had difficulties with fatigue (that is, felt very tired)].

### Defining Traumatic Encephalopathy Syndrome

#### Clinical Features

The proposed TES research criteria (19) have three core clinical features (i.e., cognitive, behavioral, mood) and nine supportive features (i.e., impulsivity, anxiety, apathy, paranoia, suicidality, headache, motor signs, documented decline, and delayed onset). This study assessed all three core features and six of the nine supportive features. Given that this was a survey, self-reported cognitive impairment was used to define the core cognitive feature, not neuropsychological testing. We elected to not assess for suicidality in this survey. The two clinical course criteria, documented decline and delayed onset, were not assessed because this community-dwelling sample of middle-aged men were not presenting to a healthcare professional for assessment or treatment.

To meet the proposed criteria for TES, participants need to have one *or more core clinical features* as well as two *or more supportive features* (referred to as the TES definition). To fulfill the criteria for a TES feature, the participant had to endorse at least one symptom related to that criterion (see Table 2) at a frequency of “sometimes,” “often,” or “always.” We deemed this to be “mild or greater” severity. We also calculated the proportion of the sample that met each feature at “moderate” severity level by only coding those who endorsed a symptom of that feature at a frequency of “often” or “always.” Additionally, we present the proportion of the sample that endorsed one or more core clinical features as well as one or more supportive features to simulate the assumption that at least one of supportive criteria relating to the course of the clinical condition, the documented decline in functioning criterion or the delayed onset criterion, would be met in most individuals who present for clinical assessment related to potential TES/CTE. This is referred to as the Liberal TES definition. All men recruited for this study were between the ages of 35 and 55, so, in essence, they could be assumed to meet the “delayed onset” criterion if their symptoms developed after their exposure to contact and collision sports and their prior concussions. We do not know, however, how long they have been experiencing symptoms and problems.

**Table 2:** Endorsement of each question used to assess traumatic encephalopathy syndrome (TES) features as defined by Montenigro et al. (19).

			**Met neurotrauma exposure criterion**	
**TES criterion**	**Question**	**Total sample (*N =* 409)**	**No (*N =* 344)**	**Yes (*N =* 65)**
**Core clinical features (1 or more necessary)**				
Cognitive	I have noticed difficulty or problems with my ability to concentrate when reading or when working.	43.8%	40.4%	61.5%
	I have noticed difficulty or problems with my memory.	38.6%	35.2%	56.9%
	I have noticed difficulty or problems with my ability to think logically or to solve problems.	23.2%	20.6%	36.9%
Behavioral	I felt angry.	45.5%	43.3%	56.9%
	I felt like I was ready to explode.	22.7%	19.8%	38.5%
	I have had problems controlling my anger.	19.1%	16.9%	30.8%
	I have been “emotionally explosive,” such as having a “short fuse” or being “out of control.”	17.1%	14.8%	29.2%
Mood	I have felt sad.	50.6%	50.0%	53.8%
	I felt depressed.	43.8%	43.0%	47.7%
	I have felt hopeless.	36.2%	34.0%	47.7%
**Supportive features (2 or more necessary)**				
Impulsivity	I have engaged in excessive gambling.	6.1%	4.7%	13.8%
	I have engaged in what some people would consider increased or unusual sexual behavior.	14.7%	14.0%	18.5%
	I have engaged in excessive shopping or unusual purchases.	11.5%	9.6%	21.5%
Anxiety	I felt anxious.	48.7%	48.0%	52.3%
	I felt worried.	53.3%	52.9%	55.4%
	I felt nervous.	45.7%	45.6%	46.2%
	I have had thoughts that keep repeating in my mind that I wish would go away.	36.2%	34.6%	44.6%
Apathy	I have had little interest in things that I used to enjoy doing.	39.1%	36.6%	52.3%
	I feel like I have had a loss of motivation and/or the experience of emotions.	39.9%	37.5%	52.3%
Paranoia	I have felt highly suspicious of other people or very jealous, even though I did not have a good reason for these feelings.	18.3%	17.2%	24.6%
Suicidality	Not assessed.	–	–	–
Headache	I have had problems with headaches (not migraines).	38.4%	34.9%	56.9%
	I have had migraine headaches.	18.6%	14.8%	38.5%
	I have had a significant problem with headaches (any type of headache) at least once per month for the past 6 months.	32.8%	28.2%	56.9%
Motor signs	I have had difficulty with my speech, such as slurred, or slow speech.	5.1%	3.2%	15.4%
	I have been moving much more slowly than in the past.	23.2%	20.6%	36.9%
	I have noticed difficulties or problems with my balance and my ability to walk.	13.0%	10.2%	27.7%
	I have lost my balance and fallen.	5.1%	4.4%	9.2%
	I have a tremor (such as hand shaking).	9.8%	8.1%	18.5%
Documented decline	Not assessed.	–	–	–
Delayed onset	Not assessed.	–	–	–
**Other health problems/Not part of TES criteria**				
Sleep Problems	I have had trouble falling or staying asleep.	69.9%	67.2%	78.5%
Chronic Pain	I have had pain in one or more parts of my body.	77.3%	74.7%	90.8%

*Symptoms were endorsed as “sometimes,” “often,” or “always” over the past year. The repetitive neurotrauma exposure criteria for TES are (i) experiencing 4 or more lifetime concussions or (ii) playing contact or collision sports for 6 or more years, including at least two at the collegiate level*.

#### History of Multiple Impacts to the Head

The neurotrauma exposure criterion for TES published in Montenigro et al. (19) is broad and diverse, and it includes three “types of injuries” arising from four “sources of exposures” (19). The three types of injuries are: (i) four or more concussions/mild TBIs; (ii) two or more moderate or severe TBIs; or (iii) “subconcussive trauma.” The sources of exposure are: “(i) involvement in ‘high exposure' contact sports (including, but not limited to, boxing, American football, ice hockey, lacrosse, rugby, wrestling, and soccer) for a minimum of 6 years, including at least 2 years at the college level (or equivalent) or higher; (ii) military service (including, but not limited to, combat exposure to blast and other explosions as well as non-combat exposure to explosives or to combatant training or breach training); (iii) history of any other significant exposure to repetitive hits to the head (including, but not limited to, domestic abuse, head banging, and vocational activities such as door breaching by police), or (iv) for moderate or severe TBI, any activity resulting in the injury (e.g., a motor vehicle accident)” [(19), p. 10–11].

We used the above criteria published by Montenigro et al. (19) to define subgroups in some of our analyses in this study. To explore the proportion of the sample who met criteria for these different neurotrauma thresholds, we formed neurotrauma exposure subgroups as follows: (a) 4 of more prior concussions/MTBIs (consistent with injury type i above); (b) 6 or more years playing a “high exposure” contact sport (i.e., American football, ice hockey, lacrosse, rugby, wrestling, and soccer) at the high school level or higher; (c) 6 or more years playing a “high exposure” contact sport at the high school level or higher, including at least 2 years at the college level or higher (consistent with “subconcussive trauma” of injury type iii, with source of exposure i, above); (d) 4 or more concussions *or* 6 or more years playing a “high exposure” contact sport at the high school level or higher, including at least 2 years at the college level or higher (the combination of injury types i and iii above).

### Statistical Analyses

Data analyses were completed in SPSS 24.0. We present the proportion of the sample who reported each core clinical feature and each supportive symptom feature. Further, we report the proportion of the sample who met these features based on their repetitive neurotrauma exposure history (e.g., those with 0–3 prior concussions/MTBIs, those with 4+ prior concussions/MTBIs, those with 0–5 years of contact sport exposure, and those with 6+ years contact sport exposure). We present the rates of positive TES screens at mild or greater and moderate or greater severities. Chi-squared tests were used to examine the proportion of the sample that met criteria for TES across different groups (e.g., those with 0–3 prior concussions/MTBIs vs. those with 4+ prior concussions/MTBIs; those who met any neurotrauma exposure criteria vs. those who met no neurotrauma exposure criteria).

A binary logistic regression was used to assess which independent variables predicted the presence of mild or greater TES as well as moderate or greater TES. The following variables were selected *a priori* as predictors: age, history of multiple impacts to the head criteria met (i.e., the neurotrauma exposure criterion, operationally defined as either 4 or more concussions, *or* 6 or more years playing contact sports with 2 or more years at the college level or higher; binary, 1 = yes, 0 = no), moderate or greater sleep difficulties over the past year (binary; 1 = yes, 0 = no), moderate or greater pain over the past year (binary, 1 = yes, 0 = no). We chose to examine the neurotrauma exposure criterion variable as a binary variable because that is how it would be used for case identification as outlined by Montenigro et al. (19).

## Results

### Description of the Sample

A total of 435 middle-aged men completed this study. Sixteen participants were excluded because of their responses on validity items. Further, ten participants were excluded from all analyses because they reported experiencing a concussion within the past 1 year. The final analyses included 409 participants. Their mean age was 45.1 years (SD = 6.0, range = 35–55). The sample was predominantly white/Caucasian (85.6%; Black/African American = 5.6%; Asian/Asian-American = 3.7%; American Indian/Alaska Native = 1.0%; multiracial = 3.7%). About half the sample was currently married (married = 49.4%, never married = 32.3%; separated/divorced = 7.3%; living with partner = 10.0%; widowed = 4.0%). Regarding concussion history, 43.0% of the sample denied a lifetime history of any concussions (*n* = 176), while 25.2% (*n* = 103) reported 1 prior concussion, 14.7% (*n* = 60) reported 2 concussions, 6.6% (*n* = 27) reported 3 concussions, and 10.5% (*n* = 43) reported 4 or more concussions (Md = 1; IQR = 0–2; Range = 0–20). On average, participants' most recent concussion occurred 23.4 years prior to this assessment (Md = 23.2, SD = 11.2, interquartile range = 15.0–31.1).

### Individual Survey Item Endorsement Over the Past Year

As seen in Table 2, many individuals reported feeling angry (45.5%), sad (50.6%), hopeless (36.2%), anxious (48.7%), worried (53.3%), and nervous (45.7%). Regarding cognitive functioning, 43.8% reported mild or greater difficulty with concentration and 38.6% reported difficulty with memory. Headaches were commonly reported (38.4%), more so in those who met the neurotrauma exposure criterion (56.9%) than those who did not (34.9%). The majority of the sample reported mild or greater sleep difficulties (69.9%) and chronic pain (77.3%). Items related to excessive gambling (6.1%), speech difficulties (5.1%), tremor (9.8%), and losing balance and falling (5.1%) were not commonly endorsed.

### Rates of Endorsing Core Clinical Features and Supportive Features

The percentages of men in this study who met criteria for the core and supportive features of TES are presented in Table 3 (mild or greater severity) and Table 4 (moderate-severe). Approximately half the sample met criteria for each of the core clinical features at the mild level or greater (core: cognitive = 53.1%; core: behavioral = 47.4%; core: mood = 54.8%). Anxiety was the most prevalent supportive feature (65.5%), followed by apathy (47.1%), and headaches (45.5%). Paranoia was the least common supportive feature that we assessed (18.3%), although nearly one in five men in our sample endorsed some degree of paranoia. As expected, a smaller proportion of the sample met the core criteria at the severity level of moderate or worse (core: cognitive = 19.8%; core: behavioral = 11.0%; core: mood = 26.2%). These criteria are also stratified by repetitive neurotrauma history. For example, 45.0% of participants with a history of 6 or more years of contact sports met the mood criteria (mild or greater, Table 3), compared to 55.8% of participants who played 0–5 years of contact sports.

**Table 3:** Percentage of the sample that meets criteria for each traumatic encephalopathy syndrome feature at mild severity or greater, stratified by subgroups.

	***n***	**Core: Cognitive**	**Core: Behavioral**	**Core: Mood**	**Impulsivity**	**Anxiety**	**Apathy**	**Paranoia**	**Headache**	**Motor signs**
Total sample	409	53.1	47.4	54.8	24.2	65.5	47.1	18.3	45.5	30.8
Non-contact sports or no sports in high school	214	50.0	48.1	56.1	22.0	65.0	48.1	21.0	41.1	25.2
Football in high school	125	60.8	48.0	53.6	28.8	67.2	50.4	19.2	54.4	39.2
Contact sports in high school (not football)	70	48.6	44.3	52.9	22.9	64.3	40.0	8.6	42.9	32.9
0–3 Concussions	366	49.5	45.4	53.3	22.4	64.8	45.9	16.9	41.5	28.1
4+ Concussions	43	83.7	65.1	67.4	39.5	72.1	60.5	30.2	79.1	53.5
0–5 Years contact sports	369	52.3	47.4	55.8	24.4	66.4	47.4	18.4	44.2	30.6
6+ Years contact sports	40	60.0	47.5	45.0	22.5	57.5	47.5	17.5	57.5	32.5
6+ Years contact sports (with 2+ in college)	22	50.0	40.9	36.4	22.7	45.5	45.5	13.6	63.6	31.8
Did not meet neurotrauma exposure criterion	344	49.4	45.1	53.8	22.4	65.4	45.6	17.2	40.7	27.6
Met neurotrauma criterion	65	72.3	60.0	60.0	33.8	66.2	56.9	24.6	70.8	47.7
Medication for Anxiety	115	75.7	65.2	76.5	34.8	90.4	70.4	32.2	62.6	48.7
Medication for Depression	117	75.2	65.8	80.3	35.9	92.3	75.2	31.6	59.0	43.6
Medication for ADHD	37	73.0	62.2	62.2	45.9	73.0	59.5	32.4	45.9	56.8
**Of those with 0 concussions and No Contact Sports…**	126	38.1	46.8	50.0	15.9	60.3	40.5	16.7	35.7	17.5
No/Minimal pain in the past year	42	16.7	28.6	21.4	11.9	33.3	21.4	4.8	19.0	4.8
Mild or worse pain in the past year	84	48.8	56.0	64.3	17.9	73.8	50.0	22.6	44.0	23.8
No/Minimal sleep issues in the past year	55	12.7	32.7	23.6	10.9	34.5	18.2	7.3	18.2	7.3
Mild or worse sleep issues in the past year	71	57.7	57.7	70.4	19.7	80.3	57.7	23.9	49.3	25.4
Mild or worse sleep issues *and* mild or worse pain issues	61	60.7	59.0	75.4	19.7	82.0	55.7	26.2	50.8	31.1
I have felt depressed = Never or Rarely	75	21.3	30.7	16.0	12.0	38.7	17.3	5.3	28.0	6.7
I have felt depressed = Sometimes, Often, or Always	51	62.7	70.6	100.0	21.6	92.2	74.5	33.3	47.1	33.3

*Supportive criteria for suicidality, documented decline, and delayed onset were not assessed. The neurotrauma exposure criterion was operationally defined as a personal history of 4 or more concussions, 6 or more years playing contact sports with 2 or more years at the college level or higher, or both*.

**Table 4:** Percentage of the sample that meets criteria for each traumatic encephalopathy syndrome feature at moderate severity or greater, stratified by subgroups.

		**Core Clinical Features**			**Supportive Features**					
	**n**	**Cognitive**	**Behavioral**	**Mood**	**Impulsivity**	**Anxiety**	**Apathy**	**Paranoia**	**Headache**	**Motor signs**
Total sample	409	19.8	11.0	26.4	6.4	31.3	19.3	7.1	34.2	9.8
Non-contact sports or no sports in high school	214	18.7	8.9	25.7	7.5	32.7	17.8	8.4	30.8	8.9
Football in high school	125	23.2	16.0	28.0	7.2	29.6	20.0	8.0	40.8	12.0
Contact sports in high school (not football)	70	17.1	8.6	25.7	1.4	30.0	22.9	1.4	32.9	8.6
0–3 Concussions	366	17.2	7.7	23.8	5.7	29.0	16.3	6.3	30.3	7.9
4+ Concussions	43	41.9	39.5	48.8	11.6	51.2	41.9	14.0	69.8	25.6
0–5 Years contact sports	369	19.5	10.8	27.4	7.0	32.2	19.2	7.3	33.3	10.0
6+ Years contact sports	40	22.5	12.5	17.5	0.0	22.5	20.0	5.0	42.5	7.5
6+ Years contact sports (with 2+ in college)	22	27.3	9.1	18.2	0.0	13.6	18.2	4.5	45.5	9.1
Did not meet neurotrauma exposure criterion	344	16.3	7.8	24.1	6.1	30.2	16.6	6.4	29.4	8.1
Met neurotrauma exposure criterion	65	35.4	27.7	38.5	7.7	36.9	33.8	10.8	60.0	18.5
Medication for anxiety	115	29.6	20.0	47.0	9.6	60.0	32.2	14.8	51.3	16.5
Medication for depression	117	36.8	23.1	52.1	12.0	59.0	36.8	13.7	42.7	15.4
Medication for ADHD	37	45.9	21.6	32.4	10.8	51.4	27.0	16.2	40.5	8.1
**Of those with 0 concussions and No Contact Sports…**	126	15.1	7.9	20.6	7.9	29.4	11.1	8.7	24.6	4.8
No/Minimal pain in the past year	42	7.1	2.4	7.1	7.1	14.3	2.4	2.4	4.8	0.0
Mild or worse pain in the past year	84	19.0	10.7	27.4	8.3	36.9	15.5	11.9	34.5	7.1
No/Minimal sleep issues in the past year	55	7.3	1.8	5.5	7.3	10.9	1.8	3.6	10.9	1.8
Mild or worse sleep issues in the past year	71	21.1	12.7	32.4	8.5	43.7	18.3	12.7	35.2	7.0
Mild or worse sleep issues *and* mild or worse pain issues	61	23.0	14.8	34.4	9.8	42.6	19.7	14.8	41.0	8.2
I have felt depressed = Never or Rarely	75	8.0	4.0	0.0	6.7	10.7	4.0	4.0	20.0	1.3
I have felt depressed = Sometimes, Often, or Always	51	25.5	13.7	51.0	9.8	56.9	21.6	15.7	31.4	9.8

*Supportive criteria for suicidality, documented decline, and delayed onset were not assessed. Regarding medications, participants reported a past history of being recommended or prescribed medications for anxiety, depression, or ADHD. The neurotrauma exposure criterion was operationally defined as a personal history of 4 or more concussions, or 6 or more years playing contact sports with 2 or more years at the college level or higher, or both*.

### Rates of Meeting the Research Criteria for TES

**Figure 1:**
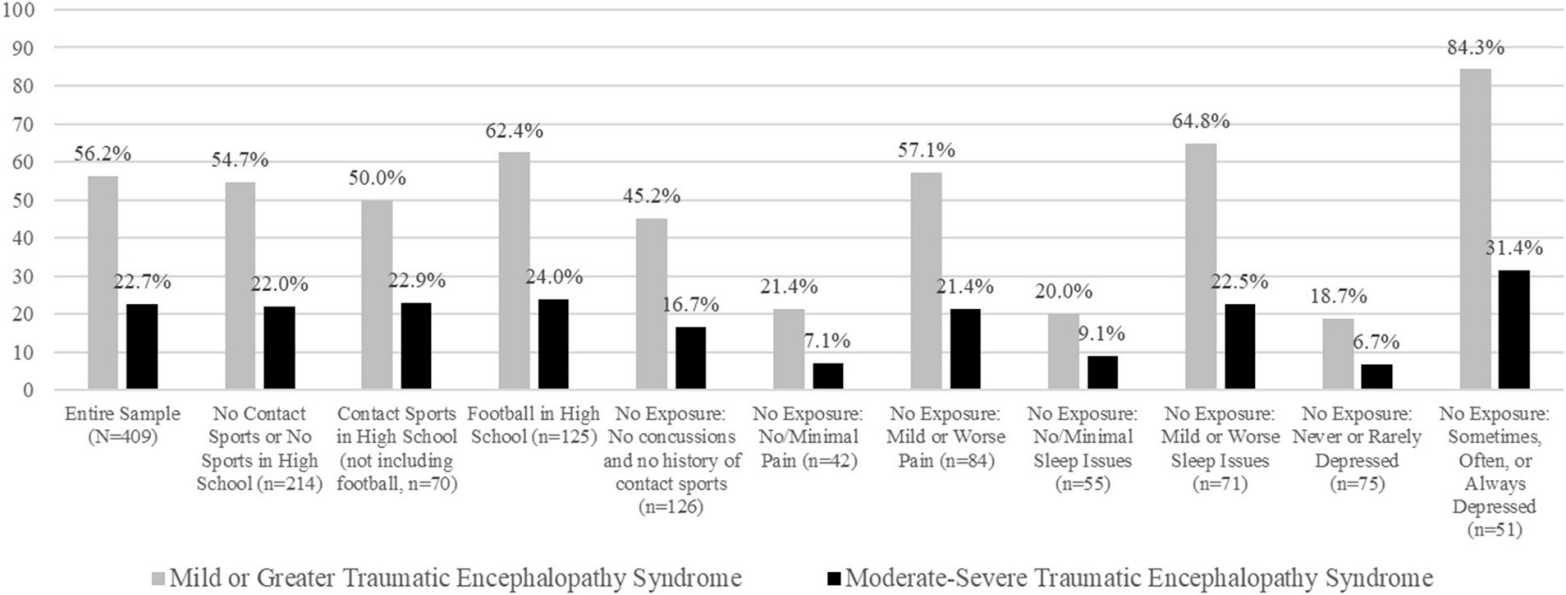
Rates of meeting research criteria for traumatic encephalopathy syndrome in men from the general population. Participants rated the frequency of their symptoms over the past year. The no exposure groups reported no history of playing contact sports and no personal history of concussion (19).

The percentage of men sampled in this study who endorsed a specific combination of symptoms that met the research criteria for TES are presented in Table 5 and visually in Figure 1. Over half of the sample met TES symptom criteria (i.e., endorsed at least one core feature and two supportive features) at mild severity or greater (56.2%), and 22.7% of participants met TES symptom criteria at moderate severity of greater. Of those with 4 or more prior concussions, 69.8% met criteria for TES compared to 54.6% of men who had 0–3 prior concussions (χ^2^ = 3.58, *p* = 0.06). About half of those with and without a 6-year history of contact sports met criteria for TES (0–5 years contact sports = 56.6%; 6+ years contact sports = 52.5%; χ^2^ = 0.25, *p* = 0.62). Results were similar for those who played 6 or more years of contact sports with at least 2 years in college (50.0%) compared to those who played 0–5 years of contact sports (56.6%; χ^2^ = 0.37, *p* = 0.55). Although we could not assess the supportive criteria related to delayed onset or progressive worsening of symptoms, it seems likely that individuals who would present to healthcare settings for their concerns, between the ages of 35 and 55 (our sample), would meet criteria for one or both of these supportive course features (e.g., the onset or progressive worsening of mental health or cognitive difficulties). Therefore, assuming one of those supportive criteria would be met, the rates of meeting the TES criteria based on one core feature and one supportive feature were 69.7% and 32.0% for mild or greater and moderate-severe, respectively.

**Table 5:** Proportion of the sample that met symptom criteria for traumatic encephalopathy syndrome, stratified by neurotrauma exposure groups, and specific symptom reporting.

**Samples**	***n***	**Mild+TES**	**Mild+TES, Liberal**	**Moderate+TES**	**Moderate+TES, Liberal**
Total sample	409	56.2	69.7	22.7	32.0
Non-contact sports or no sports in high school	214	54.7	69.6	22.0	33.2
Football in high school	125	62.4	70.4	24.0	32.8
Contact sports in high school (not including football)	70	50.0	68.6	22.9	27.1
0–3 Concussions	366	54.6	68.0	19.9	29.0
4+ Concussions	43	69.8	83.7	46.5	58.1
0–5 Years contact sports	369	56.6	69.6	23.3	32.8
6+ Years contact sports	40	52.5	70.0	17.5	25.0
6+ Years contact sports (with 2+ in college)	22	50.0	68.2	18.2	27.3
Did not meet neurotrauma exposure criterion	344	54.7	67.7	20.3	29.4
Met neurotrauma exposure criterion	65	64.6	80.0	35.4	46.2
Recommend/Prescribed medication for anxiety	115	87.0	89.6	42.6	54.8
Recommend/Prescribed medication for depression	117	84.6	88.0	43.6	57.3
Recommend/Prescribed medication for ADHD	37	75.7	78.4	35.1	48.6
**Of those with 0 concussions and No Contact Sports…**	126	45.2	62.7	16.7	27.8
No/Minimal pain in the past year	42	21.4	35.7	7.1	11.9
Mild or worse pain in the past year	84	57.1	76.2	21.4	35.7
No/Minimal sleep issues in the past year	55	20.0	41.8	9.1	10.9
Mild or worse sleep issues in the past year	71	64.8	78.9	22.5	40.8
Mild or worse sleep issues *and* mild or worse pain issues	61	67.2	82.0	24.6	42.6
I have felt depressed = Never or Rarely	75	18.7	40.0	6.7	9.3
I have felt depressed = Sometimes, Often, or Always	51	84.3	96.1	31.4	54.9

*ADHD, Attention-Deficit/Hyperactivity Disorder; TES, Traumatic Encephalopathy Syndrome. To meet the proposed criteria for Mild + TES, participants needed to have one or more core clinical features and two or more supportive features, each rated as “sometimes,” “often,” or “always.” To meet the proposed criteria for Mild + TES, Liberal, participants needed to have one or more core clinical features and one or more supportive features, each rated as “sometimes,” “often,” or “always.” To meet the proposed criteria for Moderate + TES, participants needed to have one or more core clinical features and two or more supportive features, each rated as “often” or “always.” To meet the proposed criteria for Moderate + TES, Liberal, participants needed to have one or more core clinical features and one or more supportive features, each rated as “often” or “always.” The neurotrauma exposure criterion was operationally defined as a personal history of 4 or more concussions, or 6 or more years playing contact sports with 2 or more years at the college level or higher, or both*.

### Associations With Chronic Pain and Sleep Problems

The majority of participants reported pain over the past year (never or rarely, *n* = 93, 22.7%; sometimes, *n* = 164, 40.1%; often or always, *n* = 152, 37.2%). Similarly, most individuals reported sleep difficulties occurring “sometimes” or more frequently (never or rarely, *n* = 127, 31.1%; sometimes, *n* = 157, 38.4%; often or always, *n* = 125, 30.6%). The binary logistic regression model predicting the presence of mild or greater TES was statistically significant [χ^2^(4) = 107.98, *p* < 0.001] and explained 31.1% of the variance (Nagelkerke R^2^; Table 6). Significant predictors (listed in order of magnitude from largest to smallest) were: sleep difficulties [Odds ratio (OR) = 6.68], chronic pain (OR = 3.29), and age (OR = 1.04). The neurotrauma exposure criterion was not a significant predictor of mild or greater TES (*p* = 0.81). Similarly, a binary logistic regression model predicting the presence of moderate-severe TES was also statistically significant [χ^2^(4) = 95.69, *p* < 0.001] and explained 31.7% of the variance. Significant predictors were: sleep difficulties (OR = 6.17) and chronic pain (OR = 3.09). Age (*p* = 0.54) and the neurotrauma exposure criterion (*p* = 0.20) were not significant predictors.

**Table 6:** Logistic regression predicting meeting research criteria for traumatic encephalopathy syndrome.

**Mild or greater traumatic encephalopathy syndrome**							
	**B**	**SE**	**Wald**	**df**	***p***	**OR**	**95% CI for OR**
Age	0.04	0.02	4.14	1	0.04	1.04	1.00–1.08
Neurotrauma exposure criterion	0.08	0.33	0.06	1	0.81	1.09	0.57–2.07
Chronic pain (Moderate or Greater)	1.19	0.26	20.68	1	<0.001	3.29	1.97–5.49
Sleep problems (Moderate or Greater)	1.90	0.31	37.54	1	<0.001	6.68	3.64–12.26
**Moderate or greater traumatic encephalopathy syndrome**							
Age	−0.01	0.02	0.38	1	0.54	0.99	0.94–1.03
Neurotrauma exposure criterion	0.43	0.34	1.64	1	0.20	1.54	0.79–3.00
Chronic pain (Moderate or Greater)	1.13	0.29	15.21	1	<0.001	3.09	1.75–5.44
Sleep problems (Moderate or Greater)	1.82	0.28	41.60	1	<0.001	6.17	3.55–10.72

*OR, odds ratio. The results from additional logistic regressions (not shown) were similar when including subjects who reported “mild or greater” pain or sleep instead of “moderate or greater.” The neurotrauma exposure criterion was operationally defined as a personal history of 4 or more concussions, 6 or more years playing contact sports with 2 or more years at the college level or higher, or both*.

As a secondary analysis, we examined the subgroup who reported playing high school football (*n* = 125). A binary logistic regression model predicting the presence of mild or greater TES was statistically significant [χ^2^(4) = 32.04, *p* < 0.001] and explained 30.8% (Nagelkerke R^2^) of the variance. Significant predictors were moderate or worse sleep difficulties (OR = 5.93) and moderate or worse chronic pain (OR = 3.40). Age and the neurotrauma exposure criterion were not significant predictors in the model (*p* = 0.74 and *p* = 0.41, respectively).

When analyzing the subset of the sample who did not endorse prior concussions or a history of contact sports (*n* = 126), 45.2% met criteria for mild or greater TES and 16.7% met criteria for moderate-severe TES (Table 5). When stratified by their response to these health questions, 57.1% of those with mild or worse chronic pain met criteria for mild TES compared to 21.4% of those with no/minimal pain (χ^2^ = 14.42, *p* < 0.001). Those with sleep difficulties had a higher likelihood of meeting the TES criteria than those with no/minimal sleep difficulties (64.8 vs. 20.0%; χ^2^ = 25.10, *p* < 0.001).

## Discussion

We examined the research criteria for TES in a large sample of men from the US general population. There are six important findings from this study. First, as seen in Table 2, many of the proposed symptoms of TES were reported by a large number of men who did not meet either of the neurotrauma exposure criteria, such as having problems with memory (35.2%), feeling angry (43.3%), feeling depressed (43.0%), feeling anxious (48.0%), and having headaches (34.9%). Some prior studies have reported that MTurk samples endorse greater levels of anxiety and depression compared to non-clinical samples, with medium-to-large effect sizes (27), and higher rates of social anxiety (28). This appears to be the case with our sample. Second, and strikingly, *more than half of the men surveyed*, 56.2%, met symptom criteria for having TES. Third, a history of playing contact sports was *not* significantly associated with meeting the symptom criteria for TES. There was a non-significant trend for men with a self-reported lifetime history of 4 or more prior concussions (69.8%) to have a higher rate of meeting symptom criteria for TES compared to men who had 0–3 prior concussions (54.6%, *p* = 0.06). Fourth, there was a very high rate of meeting symptom criteria for TES in men with self-reported ADHD (75.7%) (as defined by reporting a lifetime history of being prescribed medication for ADHD). Clearly, this is problematic, and the criteria might be fundamentally inappropriate for men with ADHD. Fifth, in a multivariable prediction model, sleep difficulties, chronic pain, and age were significant independent predictors of meeting symptom criteria for TES, but the binary neurotrauma criterion was not. That is, years of participation in contact sports and a prior history of multiple concussions were not associated with TES after accounting for problems with sleep, chronic pain, and a person's age. Finally, considering men who reported *no prior concussions and no history of participation in contact sports*, 45.2% met symptom criteria for TES, revealing very poor specificity and a risk for a high false positive diagnosis rate.

### Study Limitations

This study has several important limitations. First, we did not assess three of the nine supportive TES diagnostic features: delayed onset of symptoms, documented decline in functioning, and suicidality. Had we accurately and reliably assessed for those criteria, the rate in which this sample would have met criteria for TES would have been higher. For example, if we assume that either of the course supportive criteria were met (i.e., having a middle-age onset or having a progressive worsening in functioning), then 69.7% of this community sample from the US general population would meet symptom criteria for TES (see Table 5, “liberal” criteria). Second, study recruitment materials (e.g., electronic informed consent form) outlined the study's intention of assessing CTE/TES symptoms within the general public. This may have influenced some men's decision to participate and could possibly have had a negatively biasing effect on symptom reporting given the media attention and public beliefs about this topic. Third, medical, neurotrauma, and sport participation history were self-reported and could not be independently verified. Fourth, we did not collect information about socioeconomic status, employment status, or disability, and these variables are likely important and should be included in future studies and prediction models. Fifth, this sample is likely not generalizable to all middle-aged men in the United States. It should be considered a sample of convenience. As noted in Table 5, 28–29% reported they had previously been recommended or prescribed medication for anxiety or depression (anxiety: *n* = 115/409; depression: *n* = 117/409), which is consistent with some prior studies indicating that MTurk samples endorse higher levels of anxiety and depression than the general population (27, 28). Finally, the current study focused solely on men given that nearly all research to date has been with men and the current media attention surrounding former football players (mostly NFL) and potential CTE diagnoses. Future research is needed to examine the criteria with women.

### Problems With the TES Research Criteria

The research criteria represent a substantial departure from how TES was conceptualized in the twentieth century. In the past, TES was considered a *neurological* syndrome or disorder. The current criteria include features from a broad range of neurological diagnoses, and the research criteria allow TES to be diagnosed if a person is believed to have Alzheimer's disease or frontotemporal dementia, even if the clinical presentation cannot be distinguished from those diseases [(19), p. 11]. Importantly, the research criteria also include a broad range of psychiatric and behavioral features that were not considered to be part of the diagnosis during the twentieth century (e.g., depression, anxiety, and suicidality). An extraordinarily broad range of mental health problems can fulfill the criteria for either a core or supportive feature of TES, including major depressive disorder, persistent depressive disorder (dysthymia), bipolar disorder, generalized anxiety disorder, obsessive-compulsive disorder, panic disorder, specific phobias, and even substance abuse. *The current criteria, as written, allow for people with mental health problems, no neurological features, and no progressive worsening of their clinical condition, to meet criteria for TES*. Therefore, an idiopathic psychiatric disorder, or a psychiatric disorder secondary to a medical condition or neurological disease, can mimic TES.

A fundamental problem with the criteria, as written, is that the *onset and course* criteria are included as two of the nine supportive clinical features (with 7 psychiatric or neurological symptoms or signs). A person must show *only two* supportive clinical features in order to meet criteria for TES. As such, every former college athlete, who participated for 4 years in sports like football, soccer, rugby, lacrosse, hockey, or, wrestling, automatically meets the neurotrauma exposure criterion (assuming that they played any contact sport for at least 2 years during high school). Furthermore, if they develop mental health problems at any point in time between the ages of 30 and death, they automatically meet the “delayed onset” supportive criterion. As such, virtually all former athletes, and military veterans, who meet the neurotrauma exposure criterion need only meet one actual supportive clinical sign or symptom in order to fulfill the diagnostic criteria. Even more problematic is that if people are conceptualized as showing “worsening” in their clinical condition, such as a gradual worsening of their mental health (or neurological problems), then they meet a second supportive feature. In other words, by meeting two presumed *natural history* criteria (delayed onset and worsening clinical condition), the person fulfills the supportive criteria *and does not need to show any actual supportive clinical signs or symptoms*. This dramatically increases the risk for false positive diagnoses because the TES onset and course represents the natural history of many idiopathic psychiatric and neurological disorders, and, by definition, all neurodegenerative diseases (e.g., Alzheimer's disease, Parkinson's disease, and frontotemporal dementia), and thus they will mimic TES if present in former athletes and military veterans. Therefore, the TES criteria could be improved if the onset and course criteria were separated and removed from the supportive features.

Additionally, the neurotrauma exposure criteria are well-defined for former athletes but not for military veterans. As written, it appears as if active duty service members and veterans who have extensive past exposure to breaching (or “blast or other explosions”; page 10) might meet the exposure criteria for “subconcussive trauma.” There is no indication of how much exposure to breaching or to “blast and other explosions” is required, at what distance, over what time period, or whether the service member or veteran had to ever experience ill effects from a low-level blast exposure. Future research is needed to define the exposure criteria in order to apply these criteria in research studies with active duty service members and veterans.

The original authors of the TES criteria anticipated future problems and limitations, noting that (i) the population prevalence of the core and many of the supportive criteria is likely relatively high, and (ii) “it is possible to meet criteria for TES and yet have an idiopathic disorder or a situationally based condition that is unrelated to the earlier history of head impact exposure” [(19), p. 15]. The results of this study, and two prior studies, confirm those problems and limitations (23, 24). In the present study, 45.2% of men with no prior history of concussions and no prior history of participation in contact sports met symptom criteria for TES, illustrating the non-specific nature of the TES criteria and representing a striking risk for misdiagnosis.

We can anticipate very high rates of possible misdiagnosis of TES in men who are experiencing depression. Of course, it is possible that their depression is related to chronic traumatic brain injury, a neurological disease, or both. However, as seen in Table 5 and in Figure 1, 84*.3% of men who reported being depressed, who did not play contact sports and reported no lifetime history of concussion, met the symptom criteria for TES*. Moreover, all men surveyed were between the ages of 35 and 55. Thus, by definition, if they had experienced repetitive neurotrauma in their teens and 20 s, they automatically would meet the delayed onset supportive criterion. Thus, as seen in Table 5, 96*.1% would meet the symptom criteria for TES if they had the delayed onset criterion and only one additional sign or symptom criterion*. Men with depression are virtually guaranteed to meet the TES supportive clinical signs and symptoms because the supportive features of the mood subtype of TES are the exact same features that are commonly comorbid with depression in men from the general population who have no history of neurotrauma and who do not have TES: suicidality, anxiety, substance abuse (“impulsivity”), and headaches. Essentially, all former collegiate and professional contact, collision, and combat sport athletes who present for a research study or clinical evaluation, any time between the ages of 30 and death, will meet symptom criteria for TES if (i) they have developed depression and (ii) they experienced any one of the following: anxiety, suicidality, alcohol abuse, drug abuse, impulsive shopping, excessive gambling, or headaches. The TES criteria are so permissive that even if the former athlete is improving with treatment he or she can still be diagnosed with TES if the clinician (or researcher) judges that he or she would not have improved if treatment was not initiated [(19), p. 10].

Finally, it is troubling that CTE is typically described as a unique neurodegenerative disease (19, 29, 30), or *fatal* neurodegenerative disease (31), yet the research criteria for TES do not require any neurological signs or symptoms and also do not require the clinical condition to be progressive. Much additional research is needed to determine whether CTE neuropathologic change is inexorably progressive, or whether it underlies specific neurological or psychiatric problems relating to a unique neurodegenerative disease (32, 33). There are no studies establishing reasonable clincopathological correlation between the three research subtypes of TES and CTE neuropathologic change, and the consensus group that defined the preliminary criteria for the neuropathology of CTE did not address whether the pathology causes, or is clearly associated with, clinical features (34).

### Clinical Implications

The authors have firsthand knowledge, from their clinical practice working with athletes and former athletes, that some current and former athletes self-report that they, or someone they know, have been diagnosed, clinically, as having CTE. Moreover, the authors have, in their clinical practices, met with high school and college aged patients who have expressed concern about having or developing CTE—including expressing existential angst over whether they should finish college, pursue a career, get married, and have a family. The first author has spoken, on many occasions, to colleagues from across the United States, who specialize in concussion, and they have reported frequently dealing with athletes and families who have expressed concern about CTE. A recently published survey of 3,913 former NFL players revealed that 2.3% of those under the age of 60 and 3.7% of those 60 or older self-reported clinician diagnosed CTE (35). The results of this study suggest that the research criteria for TES have serious limitations for use in future studies and should not be applied in clinical practice to individual patients. Moreover, informing research subjects that they have screened positively for TES (or CTE) based on these criteria is problematic, given their potential high false positive rate, and might result in harm.

### Conclusions

This study examined the research criteria for TES and found that middle aged men who participated in contact sports were no more likely to meet criteria for the syndrome than men who played non-contact sports or no sports. It is clear from the present study that a large percentage of the US general population who are experiencing chronic pain, mental health problems, or both will meet criteria for having the symptoms described as representing TES, regardless of whether or not they have experienced multiple concussions or repetitive neurotrauma in sports. The proposed criteria for TES are extraordinarily broad, non-specific, and require thoughtful revision. There is a risk for very high rates of misdiagnosis of CTE/TES using these criteria.

## Data Availability Statement

The datasets generated for this article are not readily available because several planned studies are under way. The statistical analyses and a minimum dataset are available to qualified researchers upon request. Requests to access the datasets should be directed to GI, giverson@mgh.harvard.edu.

## Ethics Statement

The studies involving human participants were reviewed and approved by University of North Carolina at Chapel Hill and Spaulding Rehabilitation Hospital. The patients/participants provided informed consent to participate in this study.

## Author Contributions

GI conceptualized and designed the study, conducted the literature review, secured funding for the study, helped conceptualize the statistical analyses, wrote portions of the manuscript, edited drafts, and agrees to be accountable for the content of the work. ZM secured the IRB, programmed the survey, conducted the data collection, designed the database, edited drafts, and agrees to be accountable for the content of the work. DT helped conceptualize the statistical analyses, conducted the statistical analyses, wrote portions of the manuscript, edited drafts, and agrees to be accountable for the content of the work. All authors contributed to the article and approved the submitted version.

## Conflict of Interest

GI serves as a scientific advisor for NanoDX™ (formerly BioDirection, Inc.), Sway Operations, LLC, and Highmark, Inc. He has a clinical and consulting practice in forensic neuropsychology, including expert testimony, involving individuals who have sustained TBIs (including athletes and former athletes). He has received research funding from several test publishing companies, including ImPACT Applications, Inc., CNS Vital Signs, and Psychological Assessment Resources (PAR, Inc.). He has received research funding as a principal investigator from the National Football League, and salary support as a collaborator from the Harvard Integrated Program to Protect and Improve the Health of National Football League Players Association Members. DT is a consultant for REACT Neuro, Inc. The remaining authors declare that the research was conducted in the absence of any commercial or financial relationships that could be construed as a potential conflict of interest.

## References

[1] Martland HS. Punch drunk. J Am Med Assoc. (1928) 91:1103–7. 10.1001/jama.1928.02700150029009

[2] Roberts AH. Brain Damage in Boxers: A Study of the Prevalence of Traumatic Encephalopathy Among Ex-Professional Boxers. London: Pitman Medical & Scientific Publishing Co. (1969).

[3] Millspaugh J. Dementia pugilistica. US Nav Med Bull. (1937) 35:297–303.

[4] Grahmann H Ule G. [Diagnosis of chronic cerebral symptoms in boxers (dementia pugilistica & traumatic encephalopathy of boxers)]. Psychiatr Neurol. (1957) 134:261–83. 10.1159/000138743 13494597

[5] Jordan BD. Chronic traumatic brain injury associated with boxing. Semin Neurol. (2000) 20:179–86. 10.1055/s-2000-9826 10946737

[6] Erlanger DM Kutner KC Barth JT Barnes R. Neuropsychology of sports-related head injury: Dementia Pugilistica to post concussion syndrome. Clin Neuropsychol. (1999) 13:193–209. 10.1076/clin.13.2.193.1963 10949160

[7] Mendez MF. The neuropsychiatric aspects of boxing. Int J Psychiatry Med. (1995) 25:249–62. 10.2190/CUMK-THT1-X98M-WB4C 8567192

[8] Parker HL. Traumatic encephalopathy (‘Punch Drunk') of professional pugilists. J Neurol Psychopathol. (1934) 15:20–8. 10.1136/jnnp.s1-15.57.20 21610785 PMC1039021

[9] Critchley M. Punch-drunk syndromes: the chronic traumatic encephalopathy of boxers. In: Vincent C editor. Neuro-chirurgie: hommage à Clovis Vincent. Paris: Maloine (1949). p. 131.

[10] Courville CB. Punch drunk. Its pathogenesis and pathology on the basis of a verified case. Bull Los Angel Neuro Soc. (1962) 27:160–8. 14023454

[11] Johnson J. Organic psychosyndromes due to boxing. Br J Psychiatry. (1969) 115:45–53. 10.1192/bjp.115.518.45 5305282

[12] Victoroff J. Traumatic encephalopathy: review and provisional research diagnostic criteria. NeuroRehabilitation. (2013) 32:211–24. 10.3233/NRE-130839 23535783

[13] Carroll EJ. Punch-drunk. Am J Med Sci. (1936) 191:706–11. 10.1097/00000441-193605000-00014

[14] Harvey PKP Newsom Davis J. Traumatic encephalopathy in a young boxer. Lancet. (1974) 304:928–9. 10.1016/S0140-6736(74)91133-7 4138601

[15] Payne EE. Brains of boxers. Neurochirurgia. (1968) 11:173–88. 10.1055/s-0028-1095326 5729752

[16] Critchley M. Medical aspects of boxing, particularly from a neurological standpoint. Br Med J. (1957) 1:357. 10.1136/bmj.1.5015.357 13396257 PMC1974382

[17] Corsellis JAN Bruton CJ Freeman-Browne D. The aftermath of boxing. Psychol Med. (1973) 3:270–303. 10.1017/S0033291700049588 4729191

[18] Mawdsley C Ferguson FR. Neurological disease in boxers. Lancet. (1963) 282:795–801. 10.1016/S0140-6736(63)90498-7 14052038

[19] Montenigro PH Baugh CM Daneshvar DH Mez J Budson AE Au R. Clinical subtypes of chronic traumatic encephalopathy: literature review and proposed research diagnostic criteria for traumatic encephalopathy syndrome. Alzheimer's Res Ther. (2014) 6:68. 10.1186/s13195-014-0068-z. Available online at: http://creativecommons.org/licenses/by/4.0/25580160 PMC4288217

[20] Jordan BD. The clinical spectrum of sport-related traumatic brain injury. Nat Rev Neurol. (2013) 9:222–30. 10.1038/nrneurol.2013.33 23478462

[21] Laffey M Darby AJ Cline MG Teng E Mendez MF. The utility of clinical criteria in patients with chronic traumatic encephalopathy. NeuroRehabilitation. (2018) 43:431–41. 10.3233/NRE-182452 30412511

[22] Reams N Eckner JT Almeida AA Aagesen AL Giordani B Paulson H. A clinical approach to the diagnosis of traumatic encephalopathy syndrome: a review. JAMA Neurol. (2016) 73:743–9. 10.1001/jamaneurol.2015.5015 27111824 PMC4922002

[23] Iverson GL Gardner AJ. Risk of misdiagnosing chronic traumatic encephalopathy in men with depression. J Neuropsychiatry Clin Neurosci. (2020) 32:139–46. 10.1176/appi.neuropsych.19010021 31587629

[24] Iverson GL Gardner AJ. Risk for misdiagnosing chronic traumatic encephalopathy in men with anger control problems. Front Neurol. (2020) 11:739. 10.3389/fneur.2020.00739 32849206 PMC7399643

[25] Chandler J Shapiro D. Conducting clinical research using crowdsourced convenience samples. Annu Rev Clin Psychol. (2016) 12:53–81. 10.1146/annurev-clinpsy-021815-093623 26772208

[26] Gosling SD Mason W. Internet research in psychology. Annu Rev Psychol. (2015) 66:877–902. 10.1146/annurev-psych-010814-015321 25251483

[27] Arditte KA Çek D Shaw AM Timpano KR. The importance of assessing clinical phenomena in mechanical turk research. Psychol Assess. (2016) 28:684–91. 10.1037/pas0000217 26302105

[28] Shapiro DN Chandler J Mueller PA. Using mechanical turk to study clinical populations. Clin Psychol Sci. (2013) 1:213–20. 10.1177/2167702612469015

[29] Montenigro PH Corp DT Stein TD Cantu RC Stern RA. Chronic traumatic encephalopathy: historical origins and current perspective. Annu Rev Clin Psychol. (2015) 11:309–30. 10.1146/annurev-clinpsy-032814-112814 25581233

[30] Stein TD Montenigro PH Alvarez VE Xia W Crary JF Tripodis Y. Beta-amyloid deposition in chronic traumatic encephalopathy. Acta Neuropathol. (2015) 130:21–34. 10.1007/s00401-015-1435-y 25943889 PMC4529056

[31] Moszczynski AJ Strong W Xu K McKee A Brown A Strong MJ. Pathologic Thr 175 tau phosphorylation in CTE and CTE with ALS. Neurology. (2018) 90:e380–7. 10.1212/WNL.0000000000004899 29298849 PMC5791789

[32] Iverson GL Keene CD Perry G Castellani RJ. The need to separate chronic traumatic encephalopathy neuropathology from clinical features. J Alzheimers Dis. (2018) 61:17–28. 10.3233/JAD-170654 29103039 PMC5734127

[33] Iverson GL Gardner AJ Shultz SR Solomon GS McCrory P Zafonte R. Chronic traumatic encephalopathy neuropathology might not be inexorably progressive or unique to repetitive neurotrauma. Brain. (2019) 142:3672–93. 10.1093/brain/awz286 31670780 PMC6906593

[34] McKee AC Cairns NJ Dickson DW Folkerth RD Keene CD Litvan I. The first NINDS/NIBIB consensus meeting to define neuropathological criteria for the diagnosis of chronic traumatic encephalopathy. Acta Neuropathol. (2016) 131:75–86. 10.1007/s00401-015-1515-z 26667418 PMC4698281

[35] Grashow R Weisskopf MG Baggish A Speizer FE Whittington AJ Nadler L. Premortem chronic traumatic encephalopathy diagnoses in professional football. Ann Neurol. (2020) 88:106–12. 10.1002/ana.25747 32281676 PMC7383807

